# Elucidating the Role of Estrogen Effects in Leukemia: Insights from Single-Cell RNA Sequencing and Mendelian Randomization

**DOI:** 10.7150/jca.100610

**Published:** 2025-01-01

**Authors:** Jiahan Zhao, Yang Yu, Cun Liu, Ruijuan Liu, Mengxuan Sun, Jing Zhuang, Changgang Sun, Qibiao Wu

**Affiliations:** 1 College of First Clinical Medicine, Shandong University of Traditional Chinese Medicine, Jinan, 250014, China.; 2 State Key Laboratory of Quality Research in Chinese Medicine, and Faculty of Chinese Medicine, Macau University of Science and Technology, Avenida Wai Long, Taipa, 999078, Macau, China.; 3 College of Traditional Chinese Medicine, Shandong Second Medical University, Weifang, 261000, China.; 4 Department of Oncology, Weifang Traditional Chinese Hospital, Weifang, 261000, China.

**Keywords:** Estrogen, Leukemia, Single-cell RNA sequencing, Mendelian randomization, Genetic risk score

## Abstract

**Background:** Epidemiological studies have confirmed the potential role of estrogen effects in influencing the development and outcome of leukemia. Estrogen effects are increasingly attracting research interest for their potential antitumor effects beyond gynecological tumors. However, their causal relationship remains unclear.

**Methods**: In a novel approach, this study integrates single-cell RNA sequencing (scRNA-seq) with Mendelian randomization (MR) to explore the relationship between estrogen (and its receptor) and leukemia (and its related proteins). This integration showcases the uniqueness of our methodology and provides a new perspective for understanding the molecular relationship between them. Secondary analyses using genetic risk scores (GRS) were performed to further verify the robustness of the results.

**Results**: Our scRNA-seq analysis identified 14 BMMC mononuclear cell subsets, and the result showed that the estrogen receptor was implicated in leukemia. The MR results showed that there was a relationship between estradiol and leukemia inhibitory factor (β = 0.0621; *P* = 0.0229), and leukemia inhibitory factor receptor (β = 0.0665; *P* = 0.0218). The result of GRS analysis verified the MR analysis.

**Conclusions:** While both scRNA-seq and MR have yielded intriguing results, inconsistencies between these methodologies hint at a more elaborate underlying mechanism. The observed discrepancies underscore the complexity of the estrogen effects-leukemia relationship, suggesting that elucidating these interactions demands larger cohorts and enhanced sequencing depth in future studies. This research paves the way for a more nuanced understanding of the role of estrogen effects in leukemia and sets the stage for targeted therapeutic interventions.

## Introduction

Leukemia constitutes a collection of lethal blood-related malignancies characterized by the transformation of hemopoietic progenitors and the diffuse infiltration of the bone marrow, including the bone marrow and the lymphatic system. In recent years, leukemia has become one of the highest mortalities among all cancers, accounting for 2.8% of all new cancer cases and 3.4% of new cancer deaths 1-3. Different genetic perturbations drive leukemia to exist in a variety of subtypes, some of which are more prevalent in children, while cases of other types of leukemia affect adults 4,5. Previous studies have recognized a number of etiological and pathogenetic mechanisms, such as genetic disorders, metabolic abnormalities, and environmental factors, which contribute to excessive proliferation, differentiation blockade, and resistance to apoptosis of leukemia cells 6. However, the primary cause of most cases is unexplained.

Given the rising incidence of hematopoietic malignancies and the challenges associated with their treatment, investigating the risk and protective factors for blood cancers, particularly leukemias, is crucial. Epidemiological studies have revealed that males, encompassing both adults and children, exhibit a heightened susceptibility to the incidence and mortality rates associated with leukemia, which suggests a potential role of estrogen effects in influencing the development and outcome of leukemia 7-9. Previous studies have confirmed that targeting the estrogenic effects of estrogen receptors α and β holds potential as a therapeutic strategy for leukemia 10-12.

Leukemia inhibitory factor (LIF) is a pleiotropic cytokine of the interleukin-6 superfamily. LIF was initially discovered as a factor to induce the differentiation of myeloid leukemia cells and thus inhibit their proliferation 13. LIF affects multiple types of leukemia cells through its functional receptor, the leukemia inhibitory factor receptor (LIFR)^14^. LIF displays a wide variety of important functions in a cell-, tissue-, and context-dependent manner in many physiological and pathological processes, including regulating cell proliferation, pluripotent stem cell self-renewal, tissue and organ development and regeneration, inflammation, infection, immune response, and metabolism. LIF and its receptor, LIFR, are proteins deeply implicated in the pathogenesis of leukemia. The meticulous regulation of them is paramount, and any perturbation can precipitate the cascade of events leading to leukemia development and its relentless progression 15-18. Their influence on such transformative cellular dynamics positions them as keystones in the intricate mosaic of leukemia biology.

Recent technological breakthroughs in single-cell RNA sequencing (scRNA-seq) have revolutionized our ability to dissect the cellular subpopulations within tumors. This innovative approach has been instrumental in the field of cancer research, providing detailed insights into how specific cell subsets contribute to cancer initiation and progression 19,20. Concurrently, epidemiological studies have increasingly utilized Mendelian randomization (MR) to assess the causal impact of risk factors 21,22. The sophistication of MR methods has made them a preferred tool for deducing the genetic underpinnings of associations between risk factors and complex diseases, thereby enhancing our comprehension of disease pathogenesis. Furthermore, the genetic risk score (GRS) serves as a valuable metric, aggregating genetic variants to quantify the overall genetic risk 23,24. This strategy not only consolidates the findings from MR analysis but also bolsters the biological validity of our research outcomes. Despite these methodological advancements, comprehensive investigations into the connection between estrogen effects and leukemia are scarce, and the causal links and regulatory mechanisms between them remain obscure.

Although a handful of studies have begun to explore the potential of these techniques—using scRNA-seq to reveal cellular heterogeneity and MR to establish causal relationships 25,26, in the current landscape of leukemia research, this combination is still a novel approach. In this groundbreaking study, we harness the power of scRNA-seq to illuminate the cellular and molecular heterogeneity of estrogen receptor in leukemia with unprecedented clarity. By integrating MR analysis, which leverages the natural randomization of genes during meiosis, we provide robust evidence for the causal association between estrogen and leukemia risk. This sophisticated combination of methodologies offers new insights into the intricate relationship between estrogen effects and leukemia, presenting potential avenues for the development of targeted preventive and therapeutic interventions that could enhance leukemia management.

## Materials and Methods

### Study design

**Figure 1:**
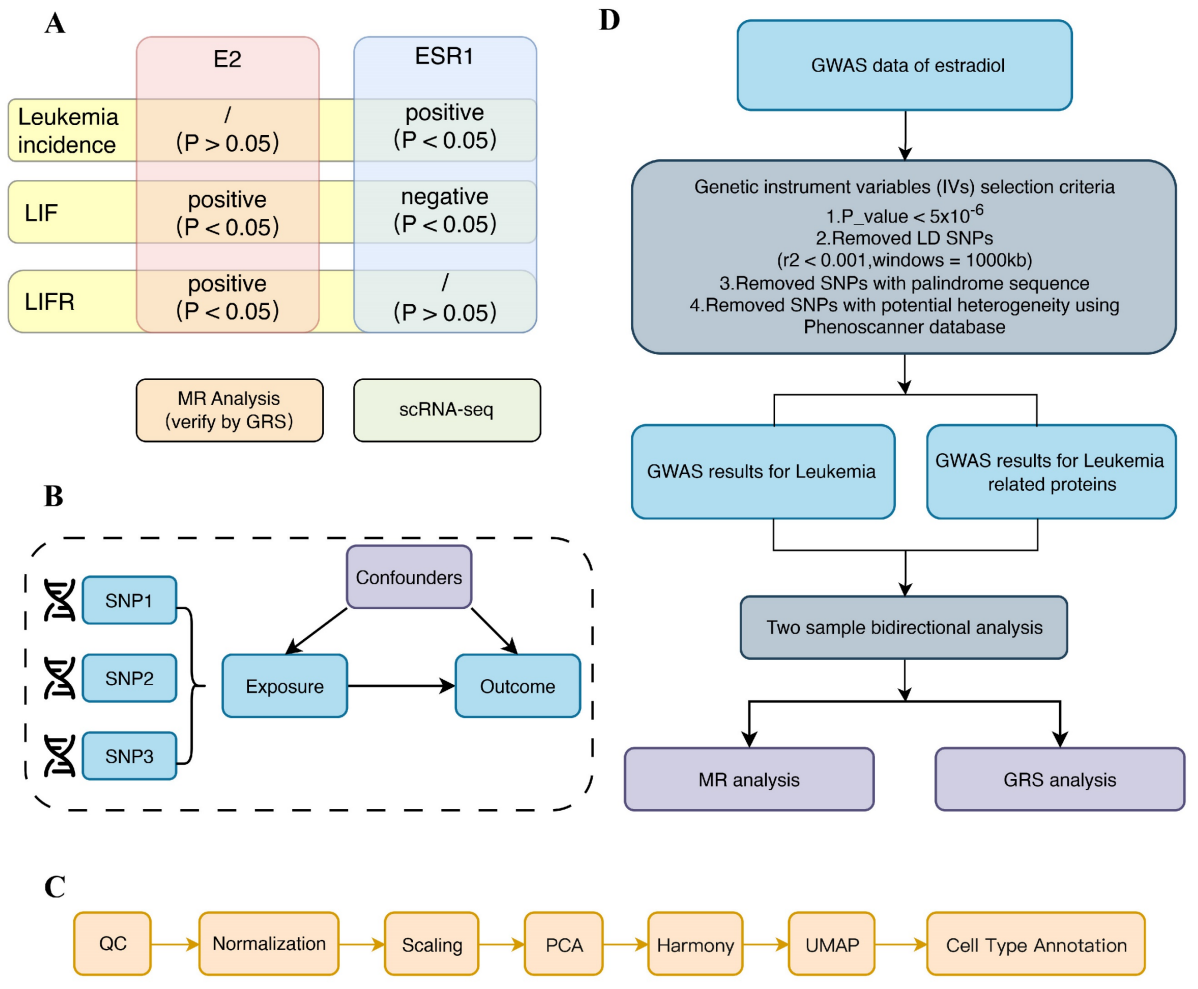
** Study design of our study.** (A) Overview of the study. (B) Hypothesis of MR analysis. (C) Flow chart of scRNA-seq. (D) Flow chart of MR analysis.

Our investigation into the relationship between estrogen effects and leukemia was conducted with utmost rigor. We combined two robust research approaches: first, we rigorously analyzed the relationship between estrogen receptor (ESR1) and leukemia using scRNA-seq. Given the central role of ESR1 in mediating the effects of estrogen, we employed ESR1 expression as a surrogate for estrogen receptor activity within the leukemia cellular microenvironment. This involved a meticulous mapping of single cells and a thorough analysis of the expression differences of related genes. Then, a two-sample MR analysis, known for its statistical power, was conducted to explore the bidirectional causality between estrogen and leukemia. To further strengthen the validity of our findings, we performed a GRS analysis. The flow diagram summarizing the methodology of the study is depicted in Figure 1.

### Acquisition of scRNA-seq data

Four samples for scRNA-seq were obtained from the Gene Expression Omnibus (GEO) database (accession number GSE139369), including two normal Bone marrow mononuclear cells (BMMC) samples and two BMMC samples from leukemia patients. Unlike the original study, we focused on estrogen receptors and leukemia-related proteins, ensuring the study's novelty of our findings.

### scRNA-seq analysis

We first set up the Seurat object (version 5.0.2) to begin the scRNA-seq analysis. Count data were normalized after quality control. Then, we identify highly variable features for the selection and scale of the data. Then, we perform linear dimensional reduction to reduce the complexity of the dataset while preserving its structure. The number of principal components involved was determined with the aid of the ElbowPlot function in Seurat. We eliminated batch effects between samples based on the top 2000 highly variable genes obtained, and Harmony was then applied. Subsequently, we run non-linear dimensional reduction techniques such as UMAP to visualize the data in a lower-dimensional space. After that, we identified cluster biomarkers by finding differentially expressed features within each cluster. Only genes that were both enriched and expressed in a minimum of 25% of the cells within at least one cell type and exhibited a log fold change exceeding 0.25, were deemed eligible for inclusion in the study. Finally, we use the “GPTCelltype” R package to assign cell type. Compared with the existing automatic and manual annotations, this annotation method has high accuracy and robustness 27, and we use “cellmarker2.0” to verify the result of its annotation. This thoroughness ensures the validity and reliability of our results.

### Differential expression analysis

We investigated the intergroup expression differences of ESR1, LIF, and LIFR, and analyzed their expression across different cell types. Additionally, we constructed pseudotime trajectory plots and illustrated the proportions of various cell types between the two groups.

### Source data of MR

The source study of exposure (estradiol) was from a generalized linear mixed model analysis 28, which applied fast GWA or fast GWA GLMM to individuals of European ancestry in the UK Biobank. They analyzed 456,348 individuals and up to 11,842,647 variants.

Data for the outcomes were obtained from two sources: the GWAS data for leukemia incidence were retrieved from the IEU OpenGWAS project dataset “ieu-b-4914”, while the genetic instruments for assessing the levels of LIF and LIFR were sourced from the INTERVAL study.

### Instrumental variable selection

Genetic IVs for estradiol, leukemia incidence, LIF and LIFR were constructed according to the following criteria 29: Only SNPs with a *P* value less than the genome-wide significance level of 5 × 10^-6^, indicating an association with disease in the respective GWAS study, were retained. Firstly, we applied a genome-wide significance threshold of *P* < 5 × 10^-8^ to filter out SNPs closely associated with estradiol, leukemia, LIF, and LIFR. However, the results indicated that the number of obtainable SNPs was limited. In such a scenario, conducting MR analysis could lead to low statistical power and weak instrument problems, resulting in biased parameter estimates 30. Therefore, we used a more liberal criterion of *P* < 5 × 10^-6^ to identify SNPs significantly associated with exposure. Then SNPs with an r^2^ > 0.001 within a 10,000 kb range of the most significant SNPs were eliminated. Allelic directions of SNPs associated with exposure and outcomes were aligned while incompatible SNPs were removed. IVs associated with potential confounding factors were further eliminated using PhenoScanner 31. Finally, to enhance the accuracy of our analysis, we applied criterion “F > 10” to filter out weak SNPs 32.

### MR analyses

MR analysis is conducted utilizing the “TwoSampleMR” R package (version 0.5.6) of the RStudio (version 4.3.2). Inverse variance weighting (IVW) was employed as the primary method of MR analysis, which is the most commonly used and mainstream method for MR analysis, uses a meta-analysis approach to combine ratio estimates of SNPs in an inverse variance weighted way and obtain an estimate of the effect of risk factors on outcomes 33,34. It ignores the presence of the intercept term in the regression and takes the inverse of the result variance as the weight for the fit, assuming that all the SNPs turn out to be valid instrumental variables and are completely independent of each other. The weighted median method incorporates weight for each value 35. A minimum of 50% of the IVs is required to be valid to obtain a robust estimate. This method tolerates more invalid IVs 36. MR-Egger regression accounts for the existence of an intercept term. It assumes that the instrument exposure and instrument outcome associations are independent-that is, the instrument strength is independent of the assumption of direct effect 37. MR-Egger regression method can provide a weighted linear regression of the outcome coefficients on the exposure coefficients and detect some violations of the standard instrumental variable assumptions and provide a non-violation-prone effect estimate 38.

### Sensitivity analysis of MR

We employed sensitivity analysis methods to assess the sensitivity of MR results, including the heterogeneity test, pleiotropy test, and leave-one-out sensitivity tests. Cochran's Q test and Rucker's Q test were used to detect the heterogeneity. If the *P* value of Cochran's Q test was less than 0.05, the final MR result referred to a multiplicative random-effects model of IVW 39. The intercept of the MR-Egger analysis results was used to test the horizontal pleiotropy 40. Additionally, the MR PRESSO method could simultaneously identify outliers and detect horizontal pleiotropy 41. We cross-validated these two horizontal pleiotropy tests to provide more robust results or correct for any pleiotropy. Finally, we performed a leave-one-out analysis and created a funnel plot to examine whether individual SNPs introduced biases into the MR results 42.

### GRS

To validate the above MR results, we conducted a secondary analysis using the GRS method, utilizing R (version 4.3.2) with the "gtx" R package (version 0.0.8 for Windows), whose "grs.summary" module contains the GRS function. The “grs.summary” module merely used single SNP association summarized data obtained from the results of the GWAS analysis, which was similar to a method that regresses an outcome onto an additive GRS 43,44.

## Results

### Comparison of Tumor Microenvironment (TME) in leukemia and normal samples

To compare TME between leukemia and normal samples, we collected four BMMC samples for scRNA-seq data. After quality control, 22595 cells were retained for subsequent analysis, including 10010 cells from the leukemia sample and 12585 cells from the normal sample.

**Figure 2:**
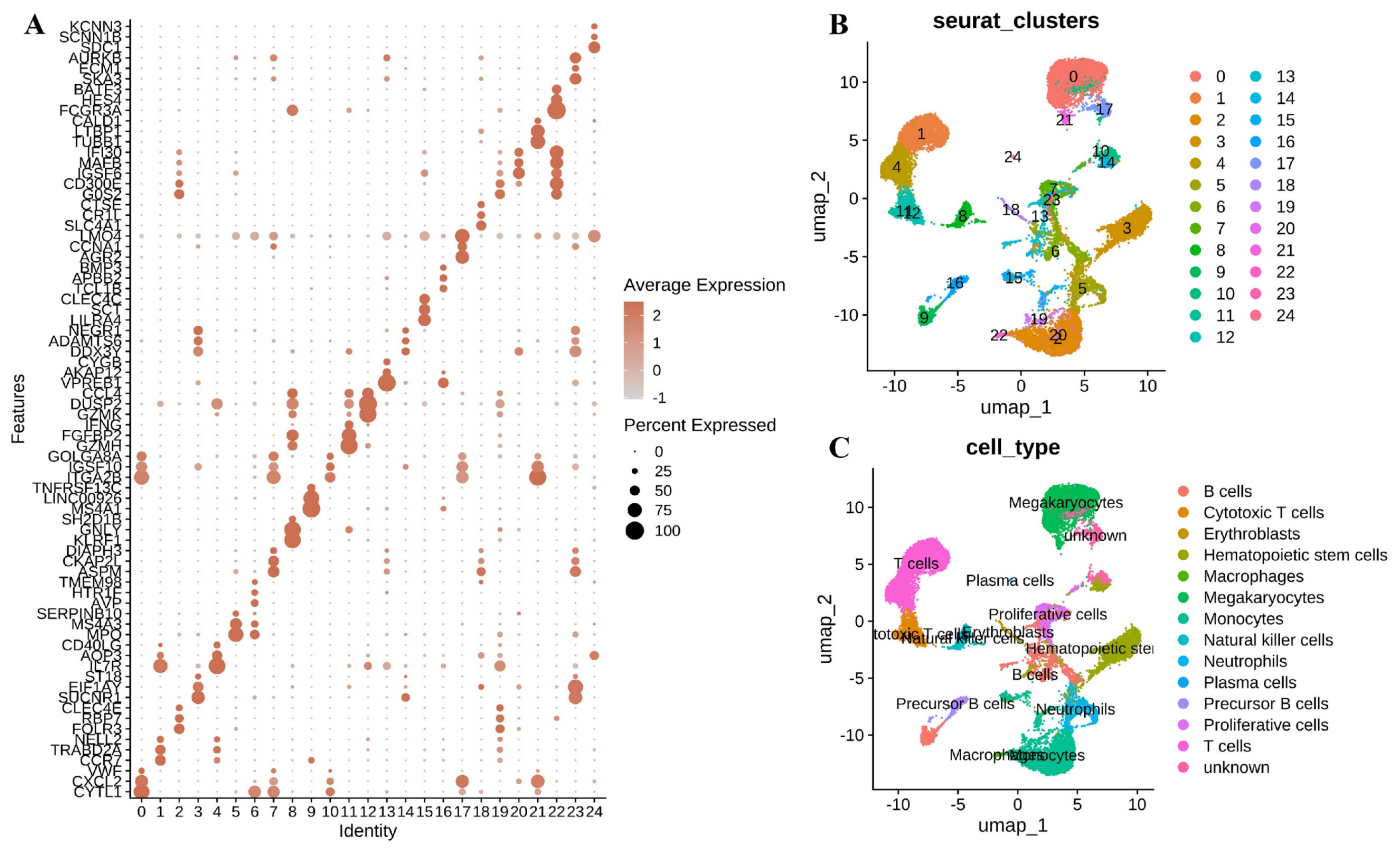
**Characterization of BMMC in normal and leukemia samples.** (A) Expression of marker genes for BMMC clusters. (B) UMAP plot of BMMC, colored by cluster. (C) UMAP plot of BMMC, colored by cell type.

We visualized the marker genes across these single-cell subpopulations within different cell clusters using a bubble plot (Figure 2A). UMAP revealed 25 distinctive cellular clusters within the BMMC context (Figure 2B). These clusters were further categorized into 14 cell types based on the expression patterns of marker genes associated with various cell lineages, including Megakaryocytes, T cells, Monocytes, Hematopoietic stem cells, Neutrophils, B cells, Proliferative cells, Natural killer cells, Cytotoxic T cells, Precursor B cells, Erythroblasts, Macrophages, and Plasma cells (Figure 2C).

### Heterogeneity of gene expression in BMMC

**Figure 3:**
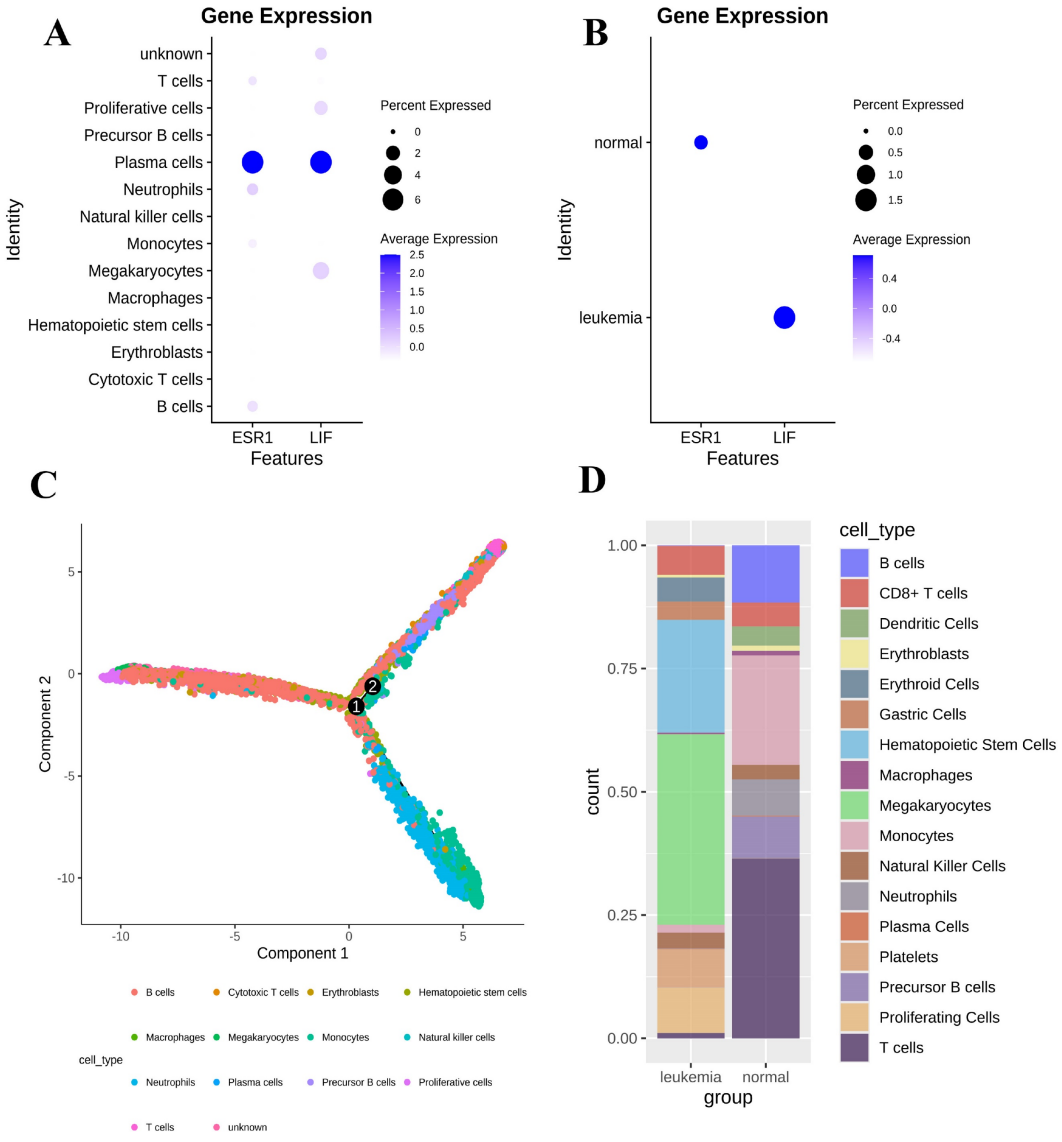
** Heterogeneity of gene expression in BMMC.** (A) Gene expression differences between cell types. (B) Gene expression differences between normal and leukemia groups. (C) Pseudotime analysis of BMMC. (D) Proportions of cell clusters in the normal and leukemia groups.

Cell clustering results showed that the ESR1 gene and LIF were mainly expressed in plasma cells (Figure 3A). We found that the expression of the ESR1 gene was higher in the normal group compared to the leukemia group, while the expression of the LIF gene was lower in the normal group compared to the leukemia group (*P* < 0.05). The LIFR gene was not detected in either group (Figure 3B). According to the scRNA-seq analysis, ESR1 expression was negatively correlated with leukemia, whereas LIF expression was positively correlated with leukemia. Then we performed a pseudotime analysis using the “monocle2" R package to better understand the progression of leukemia in the BMMC (Figure 3C). Furthermore, the cell proportion plot revealed substantial heterogeneity in the proportions of different cell types between the disease and normal groups (Figure 3D).

### MR results

**Figure 4:**
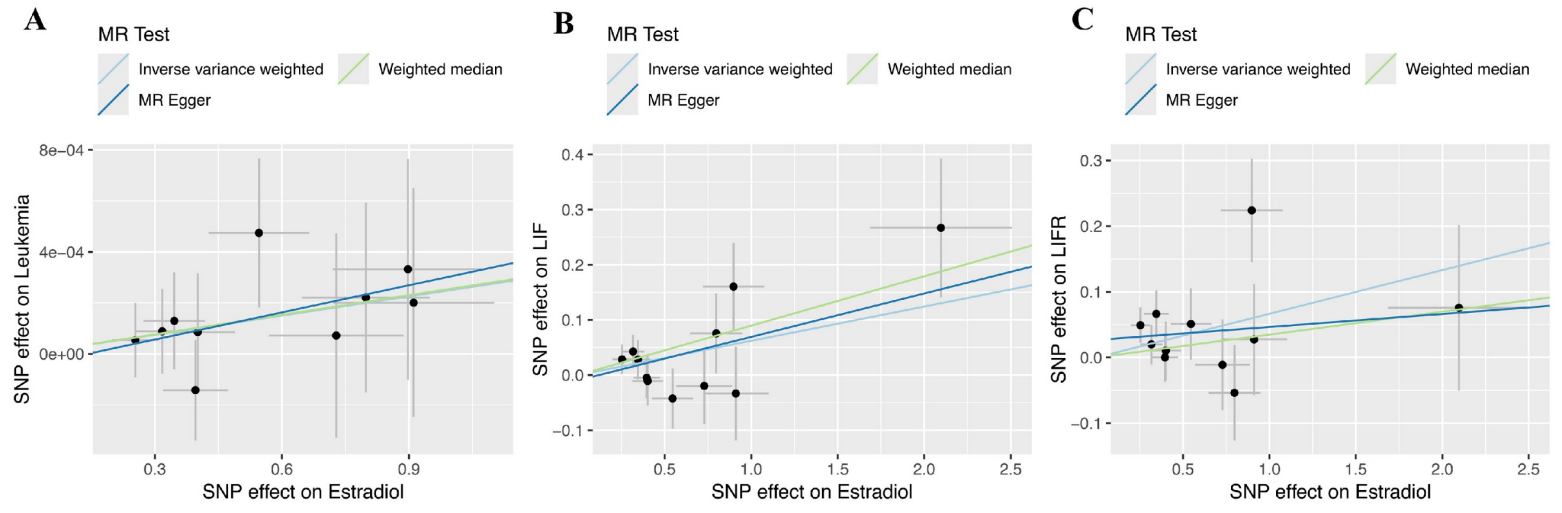
** Scatterplots of the genetic IVs association between estradiol and leukemia and its associated proteins.** (A) Leukemia incidence. (B) LIF. (C) LIFR.

**Figure 5:**
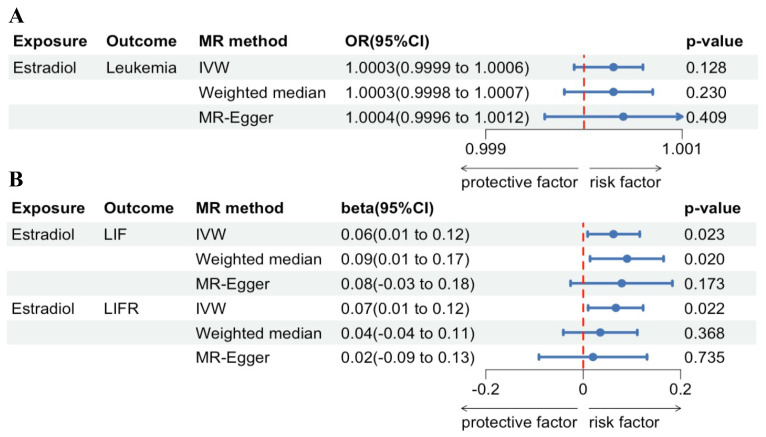
** MR analysis forest plot of estradiol on leukemia and its associated proteins.** (A) Leukemia incidence. (B) LIF and LIFR.

Regarding forward-direction MR, the estradiol to leukemia incidence MR study showed no significant association (IVW: OR = 1.0003, 95% CI = 0.9999-1.0005, *P* = 0.1278; Weighted Median: OR = 1.0003, 95% CI = 0.9998-1.0007, *P* = 0.2412; MR-Egger: OR = 1.0004, 95% CI = 0.9996-1.0012, *P* = 0.4092). In the estradiol to LIF MR study, IVW analysis and weighted median methods revealed a significant association, but MR-Egger showed no significant association (IVW: β = 0.0621, 95% CI = 0.0086-0.1156, *P* = 0.0229; Weighted Median: β = 0.0896, 95% CI = 0.0167-0.1625, *P* = 0.0161; MR-Egger: β = 0.0787, 95% CI = -0.0255-0.1828, *P* = 0.1728). In the estradiol to LIFR MR study, IVW analysis revealed a significant association, but weighted median methods and MR-Egger showed no significant association (IVW: β = 0.0665, 95% CI = 0.0097-0.1234, *P* = 0.0218; Weighted Median: β = 0.0349, 95% CI = -0.0411-0.1108, *P* = 0.3685; MR-Egger: β = 0.0198, 95% CI = -0.0913-0.1309, *P* = 0.7353). Scatter plots and forest plots are presented in Figure 4 and Figure 5.

Regarding reverse-direction MR, the IVW results of the estradiol to leukemia incidence were insignificant (*P* = 0.3852). The IVW results of the estradiol to LIF were insignificant (*P* = 0.8823). The results of the IVW analysis for the association between estradiol and LIFR were also not statistically significant (P = 0.0845).

### MR sensitivity analysis

Cochran's Q test and Rucker's Q test showed there was no heterogeneity. The intercept of the MR Egger analysis and the MR PRESSO method indicated that there was no horizontal pleiotropy, too. The F-statistics for each SNP was greater than 10, indicating that there were no weak IVs. Using Phenoscanner, we manually removed SNPs related to confounding factors, such as viral infections, immune abnormalities 45, chemical exposure 46, ionizing radiation exposure 47, and alcohol consumption 48. The results of the leave-one-out analysis and the funnel plot are provided in Supplemental Figure S1-S2. Detailed information about the SNPs used in the MR analysis can be found in Supplementary Table S1-S4.

### GRS analysis

The results of GRS analysis confirmed the causal relationship obtained by MR analysis, and the specific GRS analysis values are shown in Table 1.

## Discussion

Previous studies and epidemiological evidence have suggested a possible association between estrogen effects and leukemia 7-9. However, studies with higher levels of evidence are lacking, and little is known about the causal relationship and molecular mechanisms. In this thesis, we complemented these two approaches with scRNA-seq and MR analysis studies to explore the relationship between estrogen effects and leukemia.

Estrogen receptors are not limited to females but are present in all vertebrates, where they are involved in various physiological and pathological states 49. The effects of estrogen are influenced by both estrogen and estrogen receptors 50. Either an increase in estrogen or an increase in estrogen receptors potentiates the effects of estrogen.

Initially, we aimed to explore the relationship between estrogen, estrogen receptors, and leukemia using scRNA-seq analysis. While scRNA-seq is excellent at dissecting transcriptional heterogeneity and identifying cell types within complex tissues, it has limitations. Specifically, it only studies gene expression correlations within local tissues and cannot capture interactions between different tissues. Therefore, while we can use scRNA-seq to investigate estrogen receptor relationships in leukemia cells, we cannot assess the effects of estrogen, which is primarily secreted by the ovary 51, on leukemia using this method.

Our study takes a novel approach by using Mendelian randomization combined with scRNA-seq to delve deeper into the relationship between estrogen and leukemia. This method allows us to observe the direct effect of estrogen on leukemia, unrestricted by local tissue conditions. The use of MR analysis effectively mitigates potential biases, such as confounders and reverse causation, thereby enhancing causal inference 52. By combining scRNA-seq and the Mendelian randomization method, we effectively address the inherent limitations of each approach, providing a comprehensive exploration of the correlation between estrogen (and its receptors) and leukemia (and its related proteins). ScRNA-seq offers detailed molecular information at the cellular level, while Mendelian randomization provides causal insights into relationships between genetic variants and complex traits or diseases.

As previous studies have shown, leukemia is a highly heterogeneous disease, with significant differences between patient and normal samples 53. We specifically chose to study mixed-phenotype acute leukemias, which present with features of multiple hematopoietic lineages and can demonstrate the common characteristics of myeloid leukemia and lymphoid leukemia to a certain extent 54,55. This choice of mixed leukemia for our single-cell data set is crucial for the context and relevance of our research.

As shown in Figure 1, we explored the relationship between estrogen (E2, ESR1) and leukemia (leukemia incidence, LIF, LIFR) separately. ScRNA-seq shows a significant association between estradiol receptors and leukemia, and MR analysis suggests a causal relationship between estrogen and leukemia. While both methods yielded significant findings, they also presented some inconsistencies, indicating a more complex relationship between estrogen effects and leukemia. Possibly due to the high heterogeneity of leukemia. The Mendelian randomization study, with its larger sample size, and the scRNA-seq, which was limited to four samples, may have introduced a degree of chance, leading to the observed disparities. Besides, because the Mendelian randomized dataset is primarily derived from European populations, this is a source of potential heterogeneity, which may also limit the generality of our results.

In addition to affecting LIF and LIFR, estrogen also affects the actual clinical pathogenesis of leukemia through more complex molecular actions and cellular signaling pathways. The action of cytokines is usually regulated by a complex regulatory network, including their own regulation, the expression of ligands and receptors, and the activation state of signaling pathways 56. Cytokines can influence tumor behavior and reprogram the tumor microenvironment 57. The cytokine network is intricate, with over a hundred cytokines that share receptor components and signal transduction pathways, creating complex interactions 58. Therefore, even if the expression level of LIF and LIFR themselves changes, it is possible that the association with leukemia pathogenesis may change due to the influence of other factors.

According to the genetic central dogma, from genes to proteins, there are two processes, transcription, and translation, and there are complex regulations such as epigenetics 59,60. Methods of scATAC-seq and CITE-seq are promising approaches and deserve to be included in future studies to comprehensively investigate the relationship between estrogen effects and leukemia 61-63. At the same time, due to the strong heterogeneity of leukemia, this has led to the existing studies reporting that there is a relationship between estrogen effects and leukemia. Thus, although both methods showed a significant association between them, as for clinical incidence, only scRNA-seq showed an association, whereas MR analysis studies did not show a significant causal relationship.

Our study investigates the possible involvement of estrogen (and its receptor, ESR1) and leukemia (and its related proteins, LIF and LIFR). This research aims to explore whether estrogen effects might have antitumor effects in leukemia. By understanding the role of estrogen effects, we hope to guide treatment strategies and possibly improve outcomes for certain patients. For instance, secondary leukemia following breast cancer is a common occurrence 64. It is a question worth studying whether breast cancer patients treated with estrogen therapy may experience changes in their risk of developing leukemia.

At present, the scRNA-seq and MR datasets are still limited, and the depth of scRNA-seq is not enough. We look forward to larger samples and deeper sequencing results to further understand the relationship between estrogen effects and leukemia.

## Supplementary Material

Supplementary figures and tables.

## Figures and Tables

**Table 1:** The results of the GRS

Exposure	Outcome	OR/β (95%CI)	*P* value	Exposure	Outcome	OR/β (95%CI)	*P* value
Estradiol	leukemia	1.0003 (0.9999-1.0006)	0.1278	leukemia	Estradiol	-8.7674(-43.4587, 25.9239)	0.6204
Estradiol	LIF	0.0621(0.0086, 0.1156)	0.0229	LIF	Estradiol	-0.0183(-0.2605, 0.2239)	0.8823
Estradiol	LIFR	0.0665(0.0130, 0.1201)	0.0148	LIFR	Estradiol	0.1977(-0.0269, 0.4224)	0.0845

## Abbreviations

GWAS: genome-wide association studies
GRS: genetic risk score
IVs: instrumental variables
IVW: inverse-variance weighted
LIF: leukemia inhibitory factor
LIFR: leukemia inhibitory factor receptor
Mcl-1: myeloid cell leukemia 1
MR: mendelian randomization
MR-PRESSO: mendelian randomization pleiotropy residual sum and outlier
SNP: single nucleotide polymorphism
scRNA-seq: single-cell RNA sequencing
BMMC: bone marrow mononuclear cells
E2: estradiol
