# Supplementary material for: Elucidating the Role of Estrogen Effects in Leukemia: Insights from Single-Cell RNA Sequencing and Mendelian Randomization
Source: J Cancer. 2025 Jan 1;16(3):888–97. doi: 10.7150/jca.100610 (PMC11705057; doi:10.7150/jca.100610)
Supplement: Supplementary file 1 — Supplementary figures and tables. [file jcav16p0888s1.pdf]

Supplement materials

Supplemental Table S1. Instrumental variables for estradiol (GCST90042509) in MR analysis

SNP	effect_allele	other_allele	samplesize	eaf	beta	se	pval
rs143667962	A	G	244908	0.005547	2.09656	0.409471	3.05282E-07
rs55766813	A	G	241799	0.0527732	0.54568	0.118218	3.91371E-06
rs376934289	T	G	243190	0.146036	0.34568	0.073015	2.19727E-06
rs9363212	A	T	241687	0.313645	0.25372	0.054957	3.89916E-06
rs2886053	T	C	244510	0.13186	0.39571	0.076473	2.28547E-07
rs76620729	C	A	247508	0.0243952	0.89762	0.17782	4.46675E-07
rs3103064	T	C	246877	0.0288018	0.72829	0.159437	4.92679E-06
rs117420516	A	G	247508	0.0335888	0.79802	0.150606	1.16597E-07
rs80329144	C	A	245071	0.0212204	0.91103	0.190315	1.69354E-06
rs35568976	A	G	247508	0.0934434	0.40116	0.087871	4.98835E-06
rs28786093	A	C	227045	0.216748	0.31712	0.064441	8.60623E-07

Supplemental Table S2. Instrumental variables for leukemia (ieu-b-4914) in MR analysis

SNP	effect_allele	other_allele	samplesize	eaf	beta	se	pval
rs61774562	T	G	373276	0.018283	0.00245	0.0005	9.90011E-07
rs140842287	G	A	373276	0.011916	0.0035	0.000629	2.69998E-08
rs75799094	C	A	373276	0.049274	0.00155	0.00032	0.0000012
rs191217000	T	C	373276	0.011685	0.00322	0.000644	5.39995E-07
rs113865413	G	A	373276	0.013451	0.00394	0.00067	4.09996E-09
rs187153766	A	G	373276	0.011499	0.00345	0.000665	0.00000021
rs79400272	T	C	373276	0.068612	0.00148	0.000268	3.59998E-08
rs35391537	C	T	373276	0.026392	0.00196	0.000419	2.99999E-06
rs6475613	G	T	373276	0.53603	-0.0007	0.000135	7.39997E-07
rs72794984	T	C	373276	0.243432	-0.0007	0.000157	2.69998E-06
rs115230689	T	C	373276	0.023223	0.0021	0.000446	0.0000025
rs735665	A	G	373276	0.192695	0.00112	0.00017	4.40048E-11
rs149181464	C	A	373276	0.184292	0.00089	0.000184	0.0000012
rs75325138	A	G	373276	0.013194	0.00298	0.000588	4.20001E-07
rs138569349	A	G	373276	0.01657	0.00264	0.000556	0.000002
rs1189106	A	T	373276	0.562818	-0.0006	0.000135	3.29997E-06
rs7181030	C	G	373276	0.611325	-0.0006	0.000138	4.70002E-06
rs79420756	G	A	373276	0.034003	0.00179	0.000376	0.0000021
rs78378222	G	T	373276	0.011854	0.00326	0.000634	2.80001E-07
rs2850839	T	G	373276	0.035732	0.00177	0.000369	0.0000016

Supplemental Table S3. Instrumental variables for LIF (prot-a-1736) in MR analysis

SNP	effect_allele	other_allele	samplesize	eaf	beta	se	pval
rs4286707	C	A	3301	0.24964	-0.137	0.0281	1.09648E-06
rs78554890	T	A	3301	0.00949	-0.6052	0.1314	4.16869E-06
rs75517189	G	T	3301	0.02446	-0.3936	0.0858	4.46684E-06
rs189528308	A	T	3301	0.01044	-0.6305	0.1332	2.18776E-06
rs55745081	A	G	3301	0.05809	0.2511	0.0536	2.81838E-06
rs2467071	T	G	3301	0.04582	0.2771	0.0585	2.18776E-06
rs11045334	T	C	3301	0.01854	0.4374	0.0935	2.88403E-06
rs10842297	T	A	3301	0.15089	0.1709	0.0366	3.01995E-06
rs144035110	A	G	3301	0.02005	0.4464	0.0951	2.69153E-06
rs79892005	G	A	3301	0.03973	0.3284	0.0688	1.8197E-06
rs112854398	T	C	3301	0.1173	-0.2055	0.0447	4.36516E-06
rs4795433	T	C	3301	0.50963	-0.2008	0.0243	1.38038E-16

Supplemental Table S4. Instrumental variables for LIFR (prot-a-1737) in MR analysis

SNP	effect_allele	other_allele	samplesize	eaf	beta	se	pval
rs2096394	A	G	3301	0.65323	0.128	0.0278	4.0738E-06
rs6762723	T	G	3301	0.13127	-0.195	0.0374	1.8197E-07
rs4501084	A	T	3301	0.77377	0.1367	0.0298	4.46684E-06
rs13181677	G	T	3301	0.3468	0.1243	0.0261	1.94984E-06
rs9370497	T	C	3301	0.27522	0.1287	0.0276	3.01995E-06
rs35870858	C	G	3301	0.08137	-0.2106	0.0454	3.54813E-06
rs635634	T	C	3301	0.17981	-0.2996	0.0319	6.0256E-21
rs2882553	T	C	3301	0.24557	0.1369	0.0293	2.88403E-06
rs66992945	A	C	3301	0.04745	-0.3032	0.0657	3.98107E-06
rs7225123	C	T	3301	0.48263	0.1258	0.0252	6.0256E-07
rs117796921	C	T	3301	0.0232	-0.4128	0.0895	3.98107E-06
rs854793	A	G	3301	0.21784	-0.1456	0.0299	1.09648E-06
rs4806509	T	G	3301	0.6723	0.1352	0.0288	2.63027E-06

SNP: Single Nucleotide Polymorphism identifier; **effect_allele**: Effect allele in the exposure factor; **other_allele**: Other allele in the exposure factor; **Samplesize**: Samplesize for the exposure factor; **eaf**: Effect allele frequency for the exposure factor; **se**: Standard error for the exposure factor; **pval**: p-value for the exposure factor.

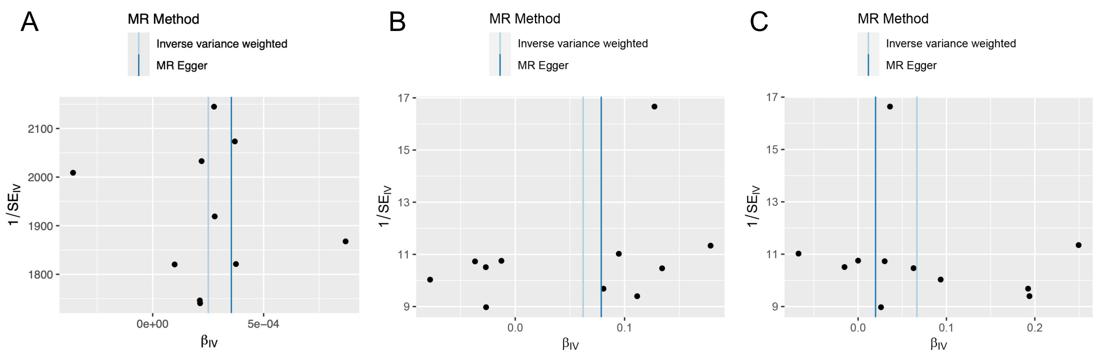

Supplemental Figure S1. Funnel plot of Mendelian randomization analysis. (A) Estradiol to leukemia incidence. (B) Estradiol to LIF. (C) Estradiol to LIFR.

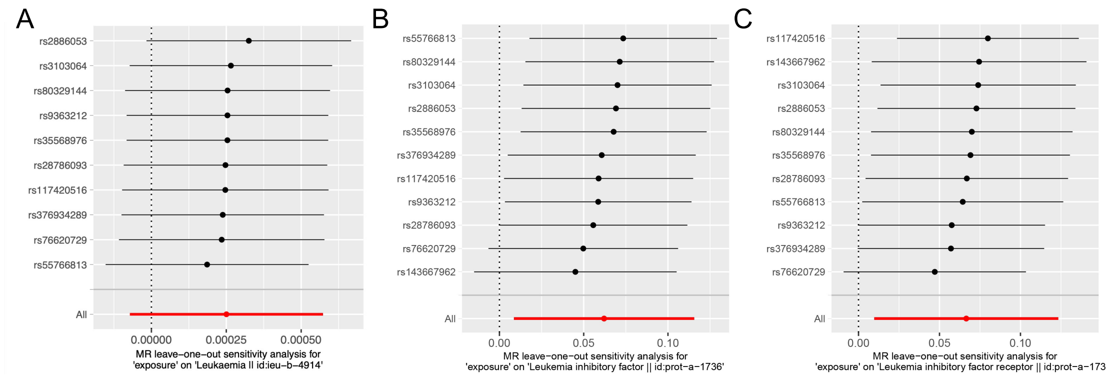

Supplemental Figure S2. Leave-one-out analysis plot in Mendelian randomization analysis. (A) Estradiol to leukemia incidence. (B) Estradiol to LIF. (C) Estradiol to LIFR.
